# Supplementary material for: Complex‐centric proteome profiling by SEC‐SWATH‐MS
Source: Mol Syst Biol. 2019 Jan 14;15(1):e8438. doi: 10.15252/msb.20188438 (PMC6346213; doi:10.15252/msb.20188438)
Supplement: Supplementary file 7 — Dataset EV6 [file MSB-15-e8438-s007.zip › feature_plots_bioplex/Q9BZW8.pdf]

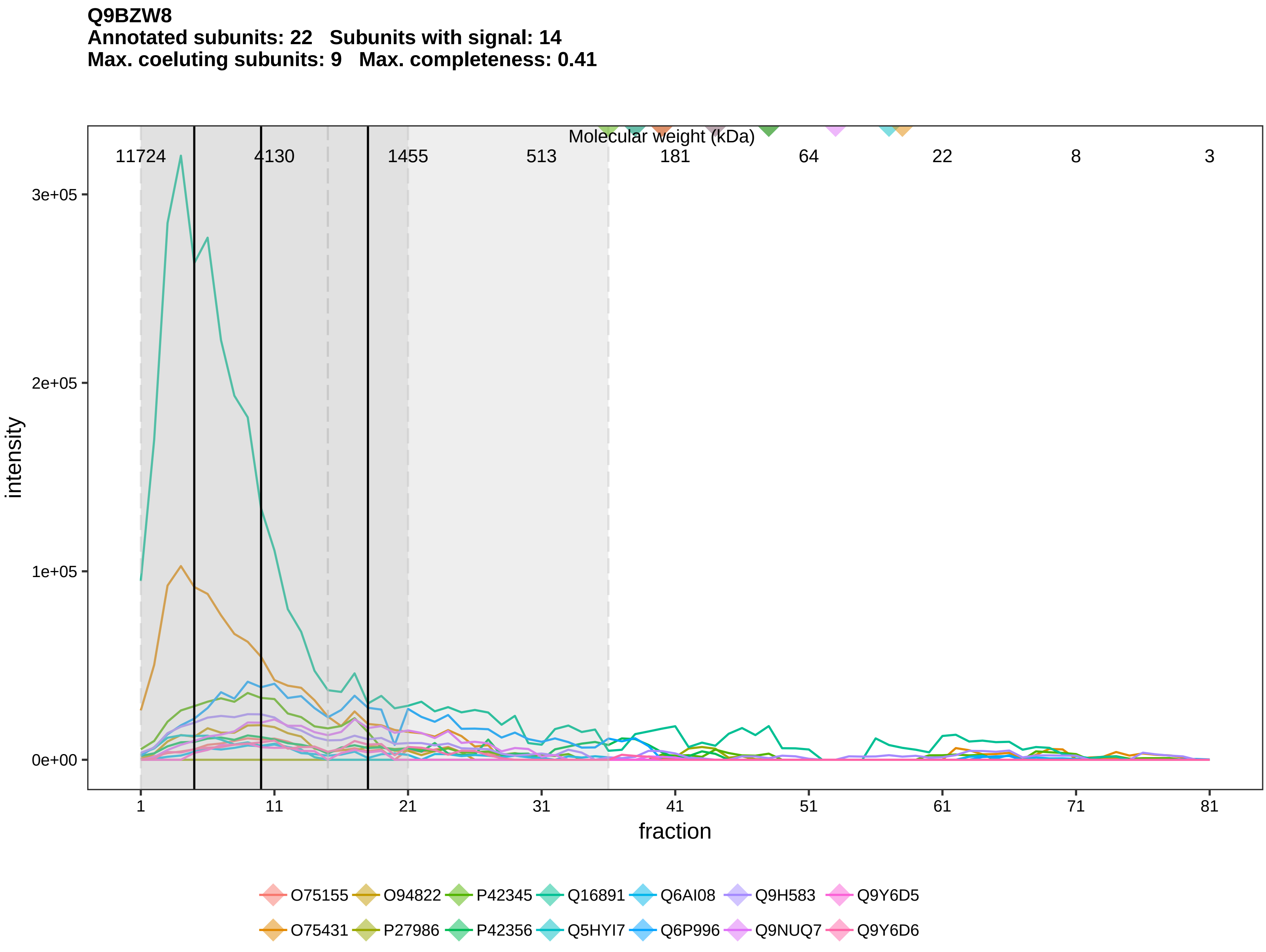
Q9BZW8
Annotated subunits: 22 Subunits with signal: 14
Max. coeluting subunits: 9 Max. completeness: 0.41
Molecular weight (kDa)
11724
4130
1455
513
181
64
22
8
3
intensity
3e+05
2e+05
1e+05
0e+00
fraction
1
11
21
31
41
51
61
71
81
O75155
O94822
P42345
Q16891
Q6AI08
Q9H583
Q9Y6D5
O75431
P27986
P42356
Q5HYI7
Q6P996
Q9NUQ7
Q9Y6D6
